# Revisiting steroidogenesis and its role in immune regulation with the advanced tools and technologies

**DOI:** 10.1038/s41435-021-00139-3

**Published:** 2021-06-14

**Authors:** Soura Chakraborty, Jhuma Pramanik, Bidesh Mahata

**Affiliations:** 1 Indian Institute of Technology, Kanpur, India; 2 Department of Pathology, University of Cambridge, Cambridge, UK

**Keywords:** Epigenetics in immune cells, Cell death and immune response, NF-kappaB

## Abstract

Historically tools and technologies facilitated scientific discoveries. Steroid hormone research is not an exception. Unfortunately, the dramatic advancement of the field faded this research area and flagged it as a solved topic. However, it should have been the opposite. The area should glitter with its strong foundation and attract next-generation scientists. Over the past century, a myriad of new facts on biochemistry, molecular biology, cell biology, physiology and pathology of the steroid hormones was discovered. Several innovations were made and translated into life-saving treatment strategies such as synthetic steroids, and inhibitors of steroidogenesis and steroid signaling. Steroid molecules exhibit their diverse effects on cell metabolism, salt and water balance, development and function of the reproductive system, pregnancy, and immune-cell function. Despite vigorous research, the molecular basis of the immunomodulatory effect of steroids is still mysterious. The recent excitement on local extra-glandular steroidogenesis in regulating inflammation and immunity is revitalizing the topic with a new perspective. Therefore, here we review the role of steroidogenesis in regulating inflammation and immunity, discuss the unresolved questions, and how this area can bring another golden age of steroid hormone research with the development of new tools and technologies and advancement of the scientific methods.

## Introduction

**Fig. 1:**
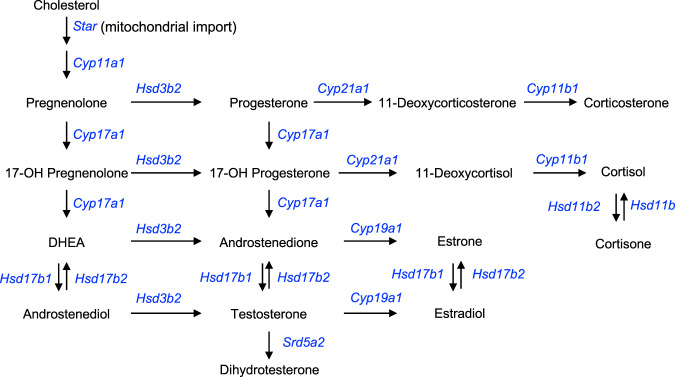
Overview of the steroid biosynthesis pathway.

**Fig. 2:**
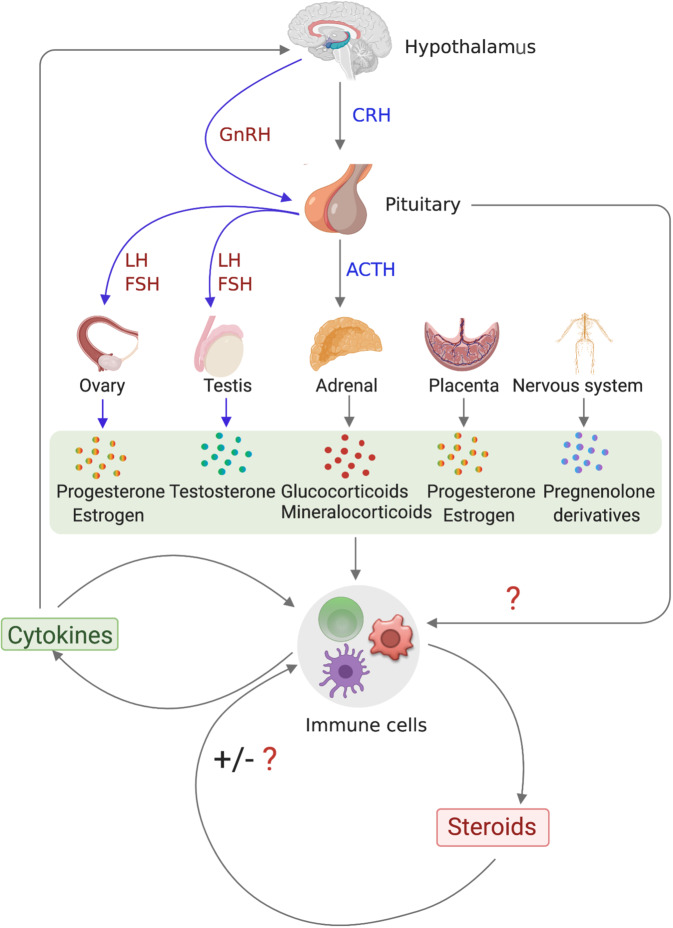
Crosstalk between steroid endocrine system and immune system. Hypothalamus–pituitary–adrenal/gonad/placenta axes regulate glandular steroidogenesis. Endocrine gland-secreted steroid hormones regulate immune-cell function. Interestingly, immune cells by themselves can synthesize steroids locally to control their own function in an autocrine and paracrine manner. A crosstalk between endocrine system and immune system exists via secretory signaling molecules. The topic needs to be revisited and further studied with modern tools and technologies. (Figure created with BioRender.com).

Cell biology and microbiology initiated in the seventeenth century with the discovery of cells and microorganisms by Robert Hooke and Antonie van Leeuwenhoek. This whole new world of life science was originated because of the technological innovations and development of practical vision-enhancing tools (e.g., optical lenses and microscopes) based on the refractive properties of glass. Similar examples are countless. Great discoveries in science originated with the advancement of tools and technologies, allowing scientists to look into the problem from diverse angles. This was not indifferent to the field of steroid hormone research. Steroidogenesis is a biosynthetic process by which cholesterol is converted into steroids (Fig. 1) [1]. Steroid hormones are synthesized mainly in the adrenal gland, gonads, and placenta under the control of the hypothalamus–pituitary–steroidogenic gland (i.e., adrenal, gonads, and placenta) axis (Fig. 2). Steroidogenesis in the other tissues, known as extra-glandular steroidogenesis (alternatively known as local steroidogenesis), in brain [2, 3], skin [4, 5], thymus [6], adipose tissues [7, 8], mucosa [9, 10] has also been reported. Interestingly, the existence of the steroidogenic immune cells, pinpointing the existence of an endogenous steroid-regulatory circuit within the immune system brought new excitement in the field (Fig. 2) [11–17]. Nevertheless, the physiological and pathological role of extra-glandular steroidogenesis remains largely unknown [18, 19]. Altogether, an important area of biology was flourished, then was temporarily neglected, but revitalizing in recent years. Here we reviewed the important discoveries of steroidogenesis and steroid regulation of immune-cell function. We raised the unanswered questions in the field and discussed new perspectives regarding how these hurdles can be overcome with the help of recent technical advancements.

## The biochemistry of steroidogenesis

The process of steroidogenesis initiates with the conversion of cholesterol to pregnenolone by cholesterol side-chain cleavage enzyme cytochrome P450scc (also known as CYP11A1, encoded by *CYP11A1* gene) within the mitochondria. Steroidogenic acute regulatory protein (StAR) facilitates the transport of cholesterol within the mitochondria [20]. Pregnenolone is then catalyzed into other steroids by a series of oxidative enzymes located in both mitochondria and endoplasmic reticulum (Fig. 1). The accessibility of these enzymes in a given tissue determines the resultant functional steroids in a given gland or tissue. The two crucial regulatory steps in this process are the transport of free cholesterol from the cytoplasm into mitochondria and conversion of cholesterol into pregnenolone. The precursor, cholesterol, comes from the cholesterol pool that are synthesized within the cell from acetate, from cholesterol ester stored in intracellular lipid droplets and from uptake of cholesterol-containing low-density lipoproteins. In chronically stimulated steroidogenic cells, plasma-borne cholesterol is the most important source. Cytochrome P450 (CYP) and hydroxysteroid dehydrogenase (HSD) are the two major classes of enzymes, which catalyzes the reactions leading to steroid biosynthesis. Typically, steroid hormones are classified into five groups: glucocorticoids, mineralocorticoids, androgens, estrogens, and progestogens. Cortisol is the major representative of glucocorticoids in mammals including humans. In rodents, it is corticosterone. Adrenal gland is the major source of glucocorticoids and mineralocorticoids. Glucocorticoids controls cell metabolism and immune-cell function. Mineralocorticoids maintain salt and water balance. Aldosterone is the most prominent mineralocorticoids. Androgens (e.g., testosterone), estrogens (e.g., estradiol and estrone), progestogens (also known as progestins) such as progesterone are synthesized by the gonads and placenta. These sex hormones control normal reproductive function. The biosynthetic pathways for major representatives of these classes of steroid hormones are illustrated in Fig. 1. It should be noted that a variety of related molecules (i.e., steroid derivatives) exist, some of which may have significant effects, but we are not discussing in this review. Pregnenolone acts as a precursor for all steroid hormones. 17α-hydroxylase hydroxylates pregnenolone and convert it to 17α-hydroxy pregnenolone. 3β-HSD oxidizes pregnenolone and forms progesterone, which is further hydroxylated by 21-hydroxylase and forms deoxy-corticosterone. All mineralocorticoids are synthesized from deoxy-corticosterone. 17α-hydroxyprogesterone is produced from progesterone or from 17α-hydroxy pregnenolone. 21-hydroxylase converts 17α hydroxyprogesterone to 11-deoxycortisol from where glucocorticoids (e.g., cortisol) are synthesized. 17, 20 lyase acts on both 17α-hydroxy pregnenolone and 17α-hydroxyprogesterone and forms dehydroepiandrosterone and androstenedione, respectively, which act as precursors for testosterone and estrogen biosynthesis.

## Steroidogenesis and steroid signaling within the immune system

Immune cells are proficient in synthesizing and metabolizing sex steroids actively [17]. Human alveolar macrophages convert androstenedione to potent androgens by catalytic activity of steroidogenic enzymes 5α-reductase, 17β-hydroxysteroid dehydrogenase (17β-HSD), 3β-HSD, and 3α-HSD. These intracellularly formed potent androgens possibly regulate the phagocytic activity of the alveolar macrophages [21]. Synovial macrophages express functional androgen receptors in both male and female. They are capable of metabolizing testosterone to active dihydrotestosterone [22]. Monocyte-derived macrophages preferentially produce a physiologically pertinent amount of androstenedione, testosterone, 3β,17β-androstenediol, 3β,16α,17β-androstenetriol, and estrogens depending on the maturation stage of macrophage and influence of local factors such as the presence of lipopolysaccharide (LPS) [23]. Monocyte and monocyte cell line THP-1 cells need to be differentiated to macrophages to produce immune reactive aromatase enzyme that catalyzes the conversion of androgens to estrogens. Dexamethasone (synthetic glucocorticoid) treated THP-1 cells exhibit upregulated activity of aromatase and also upregulation of CYP19A1 gene transcripts [24]. Nevertheless, macrophages are able to produce estrogens, which are potent local immunomodulators [25]. Androgens exhibit immunosuppressive effects, whereas estrogens have both pro- and anti-inflammatory effects depending on cell type, microenvironment, and quantity of estrogen [26]. Expression and activity of steroidogenic enzymes including 3β-HSD and 17β-HSD, 5α-reductase, aromatase have been determined in male and proestrus female mice splenic T lymphocytes after trauma-hemorrhage [27]. The study shows the involvement of intracrine sex steroid synthesis in gender dimorphic immune responses. All these steroidogenic enzymes have been found in splenic T cells. 5α-reductase activity increased in male T cells, whereas aromatase activity increased in female T lymphocytes. Increased 5α-reductase activity in male was immunosuppressive, whereas increased aromatase activity in females was immunoprotective. The immunoprotection of proestrous females enhanced reductase activity of the 17β-HSD [27]. The expression of cholesterol transporting StAR protein has been detected in mouse macrophages, which is influenced by inflammatory cytokines (tumor necrosis factor (TNF-α), IFN-γ, and TGF-β1) [28].

All these above-mentioned evidences show the ability of immune cells to convert steroids locally to another steroid. However, peripheral immune-cell-mediated steroidogenesis in true sense (also known as de novo steroidogenesis), by definition which starts with the conversion of the cholesterol into pregnenolone, was unknown until recently. In type 2 immune responses such as parasitic worm infection and Th2-infiltrated tumor microenvironment can induce de novo steroid biosynthesis in T lymphocytes, particularly in T helper 2 cells [11, 12]. In these studies, it has been proposed that type 2 immune responses elicit immune-cell steroidogenesis to resolve raised immunity and that is maladapted in solid tumors. The nature of immune-cell steroidogenesis can be context-dependent. In peanut allergy and lung airway inflammation, T-cell steroidogenesis plays a proallergic role [13–15]. Apart from T cells, macrophages can be steroidogenic in solid tumors, as is evidenced in colorectal cancer [16]. Immune-cell-mediated steroidogenesis is appearing to be conserved in human [15]. However, why type 2 immune responses evolved local immune-cell steroidogenesis, and steroid signaling is of immense importance and warrant further studies.

## Steroid regulation of macrophage function

**Fig. 3:**
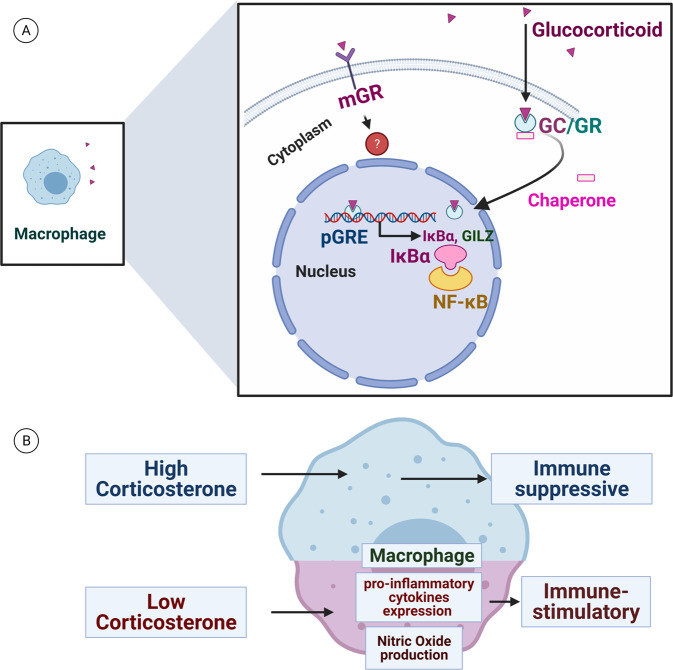
Effect of glucocorticoids (GC) on monocyte/macrophages. **A** GCs penetrate the plasma membrane and bound to the glucocorticoid receptor (GR) in the cytoplasm. The GC/GR complex is then transported to the nucleus and exert its effect by binding positive glucocorticoid response element (pGRE) or negative glucocorticoid response element (nGRE) and upregulate or downregulate the corresponding genes, respectively. Upregulated expression of IκBα impedes the effect of NF-κB. The signaling pathway through membrane-bound glucocorticoid receptor (mGR) is still unexplored. **B** GCs play contrasting roles on monocytes/macrophages in a context-dependent manner, depending upon their level and time of exposure. High level of GC is anti-inflammatory and immunosuppressive, while a low level of GC facilitates macrophage polarization into pro-inflammatory phenotypes. All these phenomena are also influenced by cytokines present in the milieu. (Figure created with BioRender.com).

Macrophages are phagocytic cells that originate from blood monocytes that leave the circulation to differentiate in different tissues. They are responsible for detecting, engulfing, and destroying pathogens and apoptotic cells, and occasionally can act as antigen-presenting cells (APCs). Steroids have miscellaneous effects on the survival and phagocytic activity of macrophages (Fig. 3). Glucocorticoids are known to impede the expansion of inflammation by suppressing pro-inflammatory macrophages as well as inducing anti-inflammatory monocyte and macrophage. Glucocorticoids penetrate the plasma membrane and bound to the glucocorticoid receptor (GR) in the cytoplasm. The glucocorticoid-GR complex then transported to the nucleus after removing the bound chaperone with GR. The glucocorticoid-GR complex can activate or repress the transcription of glucocorticoid responsive genes by binding with positive or negative glucocorticoid response elements. The liganded GR prevents the transcriptional activity of NF-κB and activator protein-1 (AP-1), which are key regulators of inflammatory response genes [29–31]. NF-κB and AP-1 may also hinder in transcriptional activation of GR. The temporal regulation of these processes in different types of tissue-resident macrophages is still unclear. Membrane-bound glucocorticoid receptors (mGR) have also been reported. Systemic lupus erythematosus patients exhibit considerably higher frequencies of mGR^+^CD14^+^ monocytes in peripheral blood, which can be downregulated by glucocorticoid treatment [32]. However, the cellular signaling through mGR is yet to be explored further in detail [33]. The lifespan of monocytes is relatively short. In the absence of any external signals, they undergo apoptosis [34]. Glucocorticoids protect anti-inflammatory monocyte/macrophages from apoptosis by specifically activating A3 adenosine receptor (A3AR) or its downstream signaling. Activation of the Raf/MEK/ERK/p90RSK pathway induces antiapoptotic effects by inhibiting caspase activity or via expressing c-Myc-dependent antiapoptotic genes, and thereby suppress inflammation [35]. Glucocorticoid-treated isolated murine macrophages are incapable of synthesizing TNF-α, which are well-known endogenous mediator for septic shock. Interferon-gamma (IFN-γ) can overcome this inhibitory effect [36]. Based on the secreted cytokines, macrophages can be classified as classically activated pro-inflammatory or M1 macrophages and alternatively activated anti-inflammatory or M2 macrophages [37]. Glucocorticoids induce differentiation of M2c macrophages [38, 39], and stimulate the survival of anti-inflammatory macrophages by upregulating and activating A3AR as an initial trigger of antiapoptotic pathway [35]. Glucocorticoids inhibit LPS/IFN-γ-induced activation (classical activation) of macrophages [40] but the effect of glucocorticoids on alternatively activated macrophages (immune complexes, adenosine receptor ligands, IL-4/IL-13 activated) and the underlying molecular mechanism is still unknown. Macrophages can convert inactive 11-dehydrocorticosterone (11-DHC) to active endogenous glucocorticoid by 11b-hydroxysteroid dehydrogenase-1 (11β-HSD1). The expression of HSD11b1 gene (encoding 11β-HSD1) is induced upon differentiation of monocyte to macrophages. Endogenous glucocorticoid is responsible for augmented phagocytotic activity of macrophages [41]. The action and regulation of this enzyme in acute inflammatory responses are yet to be explored. Macrophages destroy phagocytized microbes by producing reactive oxygen species (like nitric oxide). Corticosterone with low concentration level (10^−10^ m) shows immune-stimulatory effects by promoting the expression of pro-inflammatory cytokines and enhancing nitric oxide (NO) production in macrophages, this assists the infected organism to challenge; whereas corticosterone is immunosuppressive at higher concentration (10^−6^ m) and alters macrophage functions and protect the organism from exaggerated and harmful immune responses [42]. Glucocorticoids upregulate the expression of hemoglobin–haptoglobin scavenger receptor CD163 [43], enhance the activity of macrophages to phagocytose protein opsonized neutrophil through protein S/Mer tyrosine kinase-dependent pathway [44]. Glucocorticoids augment short term as well as prolonged phagocytosis of apoptotic cells. Short-term phagocytosis activity is enhanced by the upregulated expression of MERTK and C1q. Prolonged phagocytosis is enhanced via the induction of liver X receptors, peroxisome proliferator-activated receptors-δ, and uncoupling protein 2 (UCP2) [45]. It would be interesting to know the involvement of local steroidogenesis, particularly monocyte/macrophage-mediated steroidogenesis and steroid production, on macrophage function.

## Steroid regulation of natural killer (NK) cell function

**Fig. 4:**
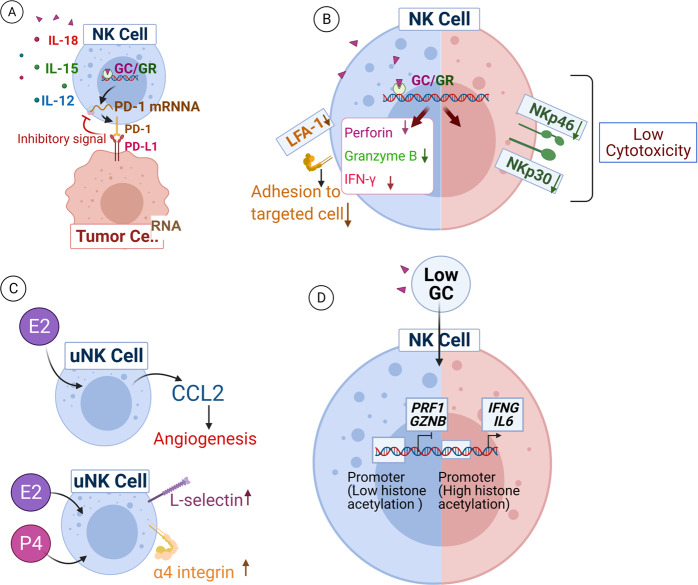
Effects of steroids on NK cells. **A** Glucocorticoids (GC) in presence of specific cytokines (IL-12, IL-15, and IL-18) induce the expression of inhibitory receptor PD-1 on CD56^bright^ NK cells, which is responsible for immunosupression in the tumor microenvironment. **B** GCs reduce the expression of perforins, granzymes, and IFN-γ by epigenetic modifications, which decrease the cytotoxic effect of NK cell. Glucocorticoids also reduce the expression of integrin lymphocyte function-associated antigen 1 (LFA-1) on the surface of NK cell, this hampers the attachment of NK cell to target cell. **C** Estradiol (E2) induces uterine natural killer cells (uNK cells) to secrete chemokine (C-C motif) ligand 2 (CCL2), which results endometrium angiogenesis. In early stage of pregnancy, estradiol (E2) or progesterone (P4) increases the expression of L-selectin and α4 integrin on circulatory CD56^bright^ NK cells. **D** Low concentration of glucocorticoids reduces histone acetylation in the promoter region of genes *PRF1* (encodes perforin) and *GZMB* (encodes granzyme B) thereby reduces the cytolytic activity of NK cells; and prime NK cells for the production of pro-inflammatory cytokines by augmenting acetylation in the enhancer or promoter region of genes *IFNG* and *IL-6*. (Figure created with BioRender.com).

NK cells are group I innate lymphoid cells. They can discriminate and destroy virus-infected cells or tumor cells based on MHC-I recognition. Specialized NK cells also play important roles in pregnancy. Glucocorticoids dysregulate NK cell function epigenetically [46]. They reduce the expression of IL-6, TNF-α, IFN-γ, granzyme B, and LFA-1. Reduced expression of integrin LFA-1 on the surface of NK cells results in decreased adhesion to the target cells [46]. Synthetic corticosteroids methylprednisolone treatment causes reduction of natural cytotoxicity receptors including NKp46, NKp30 and inhibits the expression of IL-2-inducible NKp44 receptor. Methylprednisolone-treated normal NK cells exhibit a reduced level of intra-cytoplasmic perforin, granzyme A and B [47]. Estrogen inhibits NK cell cytotoxicity, possibly through estrogen receptor (ER)-β [48]. Uterine NK (uNK) cells are the most-abundant lymphocytes in the pregnant uterus and play diverse roles including placental vascular remodeling, trophoblast invasion to establish a successful pregnancy. uNK cells lack CD16. CD16 is important for antibody-dependent cellular cytotoxicity, thus uNK cells are less cytotoxic than circulatory NK cells. In early pregnancy stage steroid hormones, 17β-estradiol or progesterone increases the expression of L-selectin and α4 integrin on circulatory CD56^bright^ NK cells [49]. These steroid hormones also induce the expression of chemokine ligands CXCL10 and/or CXCL11 on the endometrium [50]. Estradiol increases the expression of CXCR4 in uNK cells. These regulate the mobility and migration of uNK cells in the perivascular niche. Estradiol also induces uNK cells to secrete CCL2, which results in endometrium angiogenesis [51]. Dexamethasone shows differential effects on NK cells based on the local cytokine milieu. In the presence of IL-2 and IL-12, dexamethasone augments the survival and proliferation of human NK cells, increase the percentage of CD16^+^ and DNAM1^bright^ NK cells, enhance the expression of surface markers CD94 or NKG2A, and improve the mitochondrial function of NK cells, possibly by STAT4-mediated signaling. IL-2, IL-12, and dexamethasone-treated NK cells show an increased level of IFN-γ response by restimulation [52]. Low concentration of glucocorticoids reduces histone acetylation in the promoter region of genes *PRF1* (encodes perforin) and *GZMB* (encodes granzyme B), thereby reduces the cytolytic activity of NK cells. Low concentration glucocorticoid treatment prime NK cells for the production of pro-inflammatory cytokines by augmenting acetylation in the enhancer or promoter region of genes *IFNG* (encodes IFN-γ) and *IL-6* [53]. Interestingly, glucocorticoid signaling assists in host survival to mouse cytomegalovirus infection. Shortly after cytomegalovirus infection in mice, endogenous glucocorticoids produced by activation of the hypothalamic–pituitary–adrenal axis upregulate the tissue-specific expression of checkpoint receptor PD-1 on NK cells and it restricts IFN-γ production by NK cells in spleen. This prevents exaggerated lethal immune response, but does not compromise viral clearance [54]. Similar PD-1 upregulation was also observed in cancer. Glucocorticoids in combination with specific cytokines (IL-12, IL-15, and IL-18) induce the expression of inhibitory checkpoint PD-1 on CD56^bright^ subset of NK cells, the most-abundant tumor-infiltrating NK cell subset, in human. PD-L1-expressing tumor cells interact with PD-1 expressing CD56^bright^ NK cells and suppress them in the tumor microenvironment [55]. It would be interesting to know the effect of local steroidogenesis, particularly immune-cell steroidogenesis, on NK cell function in different contexts of immune responses. For example, whether the induction of local steroidogenesis in the tumor induces PD-1 expression as a negative control of antitumor immunity is unknown. The effects of steroids on NK cells are summarized in Fig. 4.

## Steroid regulation of dendritic cell (DC) function

DCs are the sentinels of adaptive immunity and immune tolerance being professional APCs. Several independent studies demonstrated that glucocorticoids induce tolerogenic phenotype in DCs [56–60]. Glucocorticoids act on DCs differentially based on the maturation stage. Dexamethasone-treated immature DCs are incapable to mature fully and unable to prime Th1 cell efficiently. Multiple restimulation of T cells with dexamethasone-treated DCs leads to the selective expansion of a specific subpopulation of T regulatory cells, Tr1, which are negative for IFN- γ and IL-4 and positive for IL-10 and do not constitutively express FoxP3 (not to confuse with so called FoxP3 expressing regulatory T cells (Treg)) [61]. On the other hand, dexamethasone treatment does not show any significant effect on LPS pre-treated DCs [62]. Prednisolone induces apoptosis in plasmacytoid DCs (pDC) after liver transplantation, and suppresses the function of toll-like receptor (TLR) stimulated pDC. TLR-stimulated pDCs are comparatively less sensitive to prednisolone-induced apoptosis. Only a higher concentration (above 1 μg/mL) of prednisolone can induce apoptosis in TLR-stimulated pDCs. Below this concentration of prednisolone (non-apoptosis inducing concentration) suppress the function of TLR-stimulated pDCs by suppressing IFN-α production and diminish their maturation to APCs. Prednisolone (non-apoptosis-inducing concentration) upregulates CCR7, CD40, CD80, and CD86 expression and also inhibit IFN-α and IL-6 production by TLR-stimulated pDC [63]. Dexamethasone-treated DCs show reduced ability to induce a primary alloreactive T-cell response and to secrete IL-Iβ and IL-12p70 [64]. Dexamethasone inhibits granulocyte-macrophage colony-stimulating factor (GM-CSF)-mediated exogenous antigen uptake and processing by airway DC cells. However, dexamethasone treatment does not influence GM-CSF-mediated upregulation of MHC-II and CTLA4 ligand expression by DC cells, thus futile in presentation of preprocessed self-antigen to alloreactive T cells in a one-way mixed lymphocyte reaction [65]. Glucocorticoids downregulate the expression of CD86, CD40, CD83, CCR7, and HLA-DR on DCs and impede IL-6 and IL-12p40 production by DCs in response to TLR7 and TLR8 agonists [66]. Corticosteroid administration to herpes simplex virus-infected patient reduces the number of IFN-α secreting pDCs in blood and IFN-α level decreases in the blood [67]. DC-secreted IL-12 is crucial for Th1 cell differentiation. Glucocorticoids inhibit IL-12 production by DCs and block the differentiation of Th1 cells. By contrast, glucocorticoids treatment augments the capacity of DCs to induce IL-4 synthesis in CD4^+^ lymphocytes. In the hyperinflammatory phase of sepsis, endogenous glucocorticoids suppress IL-12 production by DC cells and thus act as a lifeguard. Mice with knocked out GR in DCs (*Nr3c1*^fl/fl^;*Cd11c*^cre^) are highly vulnerable to LPS-induced sepsis. CD8^+^ DCs are a key source of prolonged IL-12 production in LPS-treated *Nr3c1*^fl/fl^;*Cd11c*^cre^ mice. The underlying molecular mechanism behind the exclusive sensitivity of CD8^+^ DCs to glucocorticoids is still unclear [68]. 17-β-estradiol promotes fully functional DC (in particular CD11c^+^ CD11b^intermediate^ DC population expressing high levels of MHC-II and CD86) differentiation from murine bone marrow precursors in the presence of low progesterone [69, 70]. Moreover, estradiol inhibits antigen uptake and induces the expression of pro-inflammatory cytokine genes (e.g., IL-12, IL-1, and IL-6) in CD11c^+^CD11b^intermediate^ DC. In pregnancy, elevated concentration (10^−6^ M) of progesterone reverts the effect of estradiol [70]. Altogether the current understanding is that the DCs are tolerogenic in the presence of steroids. However, it is still unknown whether immune cells produce steroids on demand to induce tolerogenic immune response of DCs to maintain homeostasis.

## Effects of steroids on T-cell development

**Fig. 5:**
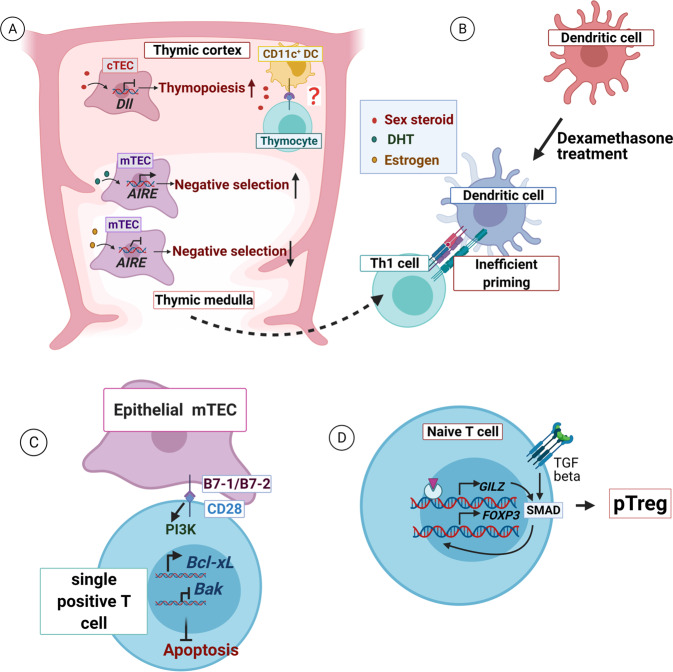
Effects of steroids on T lymphocytes. **A** Sex steroids decrease the expression of Dll in cortical thymic epithelial cells (cTEC), which results increase thymopoiesis. Dihydrotestosterone (DHT) increases the expression of AIRE in medullary thymic epithelial cells and assists in negative selection of T cells, on the other hand estrogens exhibit opposite effect by decreasing AIRE transcription. CD11c^+^ cortical dendritic cells play a crucial role in clonal deletion of activated caspase. **B** In presence of steroids DCs fail to prime T helper cells toward Th1. **C** CD28 signaling protects single positive T cells from glucocorticoid-induced apoptosis. This event exclusively occurs in later stage of thymocyte maturation. B7-1 (CD80) and B7-2 (CD86) are the ligands of CD28 are expressed in the corticomedullary region and medullary region of thymus where only single positive T cells are observed. CD28 signaling is crucial to maintain sustained expression of antiapoptotic molecule Bcl-X_L_ and downregulate the expression of pro-apoptotic molecule Bak via calmodulin and phosphatidylinositol 3 kinase (PI3K)-dependent pathway **D** Glucocorticoid-bound glucocorticoids receptor (GC/GR) induces glucocorticoid-induced leucine zipper (GILZ), which in turn induce FoxP3 expression, and thereby promoted peripheral Treg (pTreg) differentiation. (Figure created with BioRender.com).

T cells are generated from the hematopoietic stem cells of bone marrow. Progenitors then move and colonize in the thymus. Thymic stromal cells, including cortical epithelial cells in the cortex provide a suitable microenvironment essential for the T-cell differentiation and commitment [71]. Activation of δ-like (Dll) 4-Notch signaling is crucial for thymopoiesis process [72]. Sex steroid ablation results in increased expression of Dll4 and its downstream targets, which results in enhanced thymopoiesis process (Fig. 5A) [73]. Castration in male mice causes androgen deficiency, which results thymic enlargement shifting the T-cell balance toward the T helper subset and administration of androgens changing the balance toward CD8 suppressor/cytotoxic T-cell predominance [74]. The underlying mechanism is still poorly understood. Medullary thymic epithelial cells (mTECs) provide an important role in central tolerance establishment by expressing autoimmune regulator (*AIRE*) gene. AIRE induce Treg cell production and helps in the negative selection of self-reactive T cells by regulating the expression of peripheral tissue-specific antigens in mTECs. Mutation in *AIRE* gene results in multi-organ autoimmune disorders reported in mice and human [75]. Ligand-bound androgen receptor binds to the *AIRE* promoter and enhances its expression. Dihydrotestosterone administration shows increased upregulation of *AIRE* expression in human and mice thymus [76]. By contrast, estrogen downregulates *AIRE* expression by epigenetic modifications. It may be one of the reasons of sex bias in autoimmune disorders; females are more susceptible to autoimmune disorders with compare to male [77]. CD4^−^CD8^−^TCR^−^ thymocytes show the highest and CD4^+^CD8^+^TCR^low^ thymocytes show lowest expression of GR among all the developmental subsets of T cells. CD4^+^CD8^+^TCR^low^ subset of thymocytes are most sensitive to glucocorticoid-induced apoptosis [78, 79]. Whereas mature single positive T cells are resistant to glucocorticoid-induced cell death. CD28 signaling protects single positive T cells from glucocorticoid-induced apoptosis. CD28 signaling exclusively occurs in later stage of thymocyte maturation. B7-1 (CD80) and B7-2 (CD86) are the ligand of CD28 molecules and these are expressed in the corticomedullary region and medullary region of thymus where only single positive T cells are observed. CD28 signaling is crucial to maintain sustained expression of antiapoptotic molecule Bcl-X_L_ and downregulate the expression of pro-apoptotic molecule Bak in single positive thymocytes via calmodulin and phosphatidylinositol 3 kinase-dependent pathway (Fig. 5C) [80]. The role of endogenous glucocorticoids has been elegantly tested in genetically modified mouse models [81]. It was found that both mature and immature T cells are sensitive to intrinsically generated glucocorticoid-mediated apoptosis. Hydroxysteroid 11b dehydrogenase-1 enzyme (encoded by *Hsd11b1* gene) converts inactive metabolite 11-DHC into active corticosterone in thymocytes and peripheral T cells, which induces apoptosis. T-cell receptor (TCR) activation protects T cells from apoptosis (Fig. 5C) [82]. Glucocorticoid-bound GR binds to the GR motif of *Il7ra* promoter in mouse T cells [83], and influence diurnal oscillation in T-cell distribution by inducing the expression of IL-7R and CXCR4. Glucocorticoid-stimulated GR signaling augments IL-7R expression with a peak at midnight and a nadir at midday in mouse T lymphocytes. Thus, T cells accumulate in spleen and show enhanced immune responses at night [84]. The further detail study is essential to explore the diurnal rhythm-based steroid regulation of immune functions.

## Steroid regulation of T lymphocyte function

Interactions between an APC and naïve helper T cell results the differentiation of T helper cells and induction of adaptive immunity. Upon TCR activation and stimulation with lineage-determining cytokines, T helper cells differentiate into one of several lineages of T helper (Th) cells such as Th1, Th2, Th17. These differentiated T helper cells are characterized by their characteristic cytokine expression [85]. Glucocorticoids inhibit the proliferation of antigen-specific Th1 and Th2 clones and downregulate antigen-induced cytokine genes (e.g., IL-4, IL-13, and IFN-γ) expression in concentration-dependent manner. Dexamethasone also impedes antigen-induced pro-inflammatory cytokine (IL-4, IL-5, IL-13, IFN-γ) gene expression in peripheral blood mononuclear cells [86]. Dexamethasone suppresses Th1 cellular immunity by selectively impeding IL-12-induced Stat4 phosphorylation and thus suppressing IFN regulatory factor-1 promoter activity in Th1 cells [87]. GATA3 is a key transcription factor involved in Th2 humoral immune responses. Glucocorticoids inhibit the expression of GATA3 as well as cAMP‐induced PKA/p38 MAPK GATA3 phosphorylation [87, 88]. T-bet transcription factor influences the phenotype of Th1 cells and controls the expression of the potent inflammatory cytokine IFN‐γ. Glucocorticoids inhibit transcriptional activity of T-bet [89]. Sex steroids also regulate the function of T helper cells [90–92]. In contrast to the glucocorticoids, estrogens promote INF-γ expression in Th1 cells [92–94]. Estrogen-activated ER binds to the *Ifng* promoter [93, 95], and enhance the expression of transcription factor T-bet [94, 96]. However, estrogen-mediated response can be context-dependent. It can skew the immune response from Th1 to Th2 type [97–99]. The effects of estrogens on Th17 cells depend on the experimental model, leading to enhancement [100, 101] or decrease of effectiveness of these cells [102, 103]. Steroid hormones primarily suppress CD8^+^ T-cell function [104]. The activity of splenic cytotoxic T lymphocytes gets suppressed distinctly after pre-incubation with several glucocorticoids (in sub-nanomolar concentration) for several hours [104]. However, the mechanism of action, particularly how glucocorticoids effect directly on CD8^+^ cytotoxic T-cell function is surprisingly limited and still unclear. The present understanding is that the glucocorticoids activated GR trans-repress AP-1 and NF-κB transcription factors. However, recent studies raised the possibility that besides actively repressing pro-inflammatory gene expression, they may also promote suppression via transactivation of immune-suppressive genes [105]. The recent addition to this end is that the glucocorticoids may also upregulate inhibitory receptor expression in CD8^+^ T cells [16, 106]. It would be interesting to see how local T-cell-mediated steroidogenesis regulates T-cell-mediated adaptive immunity in different immunological contexts, in an autocrine and paracrine manner, particularly where Th2 cells are involved. All the previously observed suppressive effects of steroids on T cells speculate that local steroidogenesis may involve in the resolution (active termination) of immunity once T cells are done with their functional role.

## Steroid regulation of Treg

Treg, a subpopulation of suppressive T cells, are crucial for immune tolerance and essential for the maintenance of immune homeostasis. The immune-modulating capacity of these cells plays a major role in transplantation, autoimmune disease, infection, allergy, cancer, and pregnancy. Glucocorticoids regulate the development and function of these cells [107]. Glucocorticoid signaling increases both the number [107–111] and function of Treg cells [112–114]. Glucocorticoid-induced leucine zipper protein GILZ is essential for the crosstalk between glucocorticoids and TGF-β receptor (Fig. 5D). Overexpression of GILZ enhances Treg cell production, while deletion of GILZ in T cells produce fewer peripheral Treg cells [107]. Steroid regulation of Tregs is not limited to glucocorticoids. The high level of estrogen in pregnant women causes the elevation of CD4^+^CD25^+^ Treg in peripheral blood, which is responsible for immunomodulation in pregnancy [115]. Estradiol-bound ER alpha drives FOXP3 expression by binding with the FOXP3 promoter of Treg cell in both healthy male and in tumor microenvironment of cervical cancer patients [116]. It has been shown that several autoimmune disorders, including multiple sclerosis is temporally suppressed by pregnancy. In pregnant placental female, progesterone and other steroid hormones promiscuously bind to the GR in T cell and cause robust expansion and enrichment of Treg cells. This confers protection from autoimmunity in pregnant placental mammals. Further study in this field will be very effective in cure of autoimmune disorders [117]. For example, artificial induction of local steroidogenesis (e.g., immune-cell steroidogenesis) can be one such strategy. Dexamethasone increases the miR-342-3p expression in Treg cells. miR-342-3p targets rictor, a subunit of mTORC2 complex, which results in metabolic reprogramming in Treg cell through robust increase of oxidative phosphorylation [118]. FOXP3^+^CD4^+^ T regulatory cells are very delicate to dexamethasone-induced apoptosis based on dose and time of exposure to dexamethasone, but this sensitivity differs in diverse subset of FOXP3^+^CD4^+^ Treg cells. Slow-cycling Treg cells (CD25^low^CD45RA^+^) are comparatively resistant whereas fast-cycling Treg cells (CD25^high^CD45RA^−^) are relatively sensitive to dexamethasone-induced apoptosis. But once Treg cells get activated, the suppressor activity of Treg cells is not influenced by dexamethasone [119].

## Steroid regulation of B lymphocytes

After activation B lymphocytes differentiate and form memory B cell as well as antibody secreting plasma cells. B cells are also potent APCs. In mammals, B cells are originated and matured in the bone marrow. Mature B lymphocytes migrate to the secondary lymphoid organs or tissues where they interact with T lymphocytes and undergo plasmacytic differentiation. GRs have found to be highly expressed in all developmental subsets (pro/pre IgM^−^IgD^−^, immature IgM^+^IgD^−^, mature IgM^+^IgD^+^) of B220^+^ B cells. Dexamethasone administration in adrenalectomized mice causes the reduction of B-cell number in both spleen and bone marrow by inducing B-cell apoptosis [120]. Hydrocortisone induces IgE synthesis in human B cell by prompting isotype switching [121]. Glucocorticoids functionally impair upstream BCR and TLR7 signaling and significantly enhance the expression of anti-inflammatory cytokine IL-10, and terminal-differentiation factor BLIMP-1 [122]. Estrogen blocks tolerance induction of naive autoreactive B cells and upregulate the expression of antiapoptotic Bcl-2 molecule [123]. In vitro experiment on mice splenic B cell revealed that estrogen enhances the activity of B cells by downregulating CD80 expression on B cell. Decreased CD80 expression on B cell diminishes CTLA4-CD80 interaction, which impairs the negative control of T-cell activation. Estrogen upregulates IgG antibody production by splenocyte without stimulating proliferation and differentiation of B cell to plasma cell. Estrogen also protects splenic B-cell apoptosis in serum-deprived condition [124]. Testosterone is an endogenous regulator of B-cell survival factor BAFF. In castration, testosterone deficiency results in upregulation of B-cell number and increases the risk of autoimmunity [125].

## Future of steroidogenesis research with the newly developed tools and technologies

We are on the verge of a technological explosion in the area of cell and molecular biology. It is expected that many aspects of steroid signaling will be resolved in the coming years. Perhaps the most exciting area to explore would be the extra-glandular (local) steroidogenesis. During the last decades, we have seen that immune cells respond to the steroid hormone signaling. When we are beginning to better understand how steroids exert their effect on immune cells, another level of complexity and possibility arose. The existence of steroidogenesis and steroid signaling within the immune system raises new possibilities on how immune cells communicate to shape physiology of immune response and how it is maladapted in pathology. This can revolutionize the understanding of immune regulation because nuclear receptor signaling (e.g., GR, ER, androgen receptor, mineralocorticoid receptor-mediated signaling) is dramatic, and GR alone can control 20% of the genes [105, 126]. It would be exciting to discover the basic principles of immune-cell-mediated steroidogenesis in immune-cell regulation.

To discover steroid biosynthesis and metabolism pathway in immune cells, profiling and quantification of all steroids and intermediate metabolites of steroids in immune cells need to be done. The improvement in liquid chromatography/tandem mass spectrometry promises such an approach [127]. Complex steroid metabolic pathway in immune cells can be explored by metabolic flux analysis using physiologically based pharmacokinetic modeling. A recent technique called AGPathFinder can be used to find biochemically relevant metabolic pathways between two steroid metabolites [128]. The activity of steroidogenesis-specific enzymes in immune cells can be assessed with the help of chemoproteomic method activity-based protein profiling technique [129]. Discovery-based metabolomics (DMP) study can be used to identify known and unknown metabolite intermediates of steroid [130]. The fields of single-cell transcriptomics [131], multimodal omics [132], and spatially resolved [133] transcriptomics are rapidly expanding with their enormous capability. Steroidogenic and steroid-responsive gene expression and regulatory network in immune cells can be mapped applying these cutting-edge technologies. These approaches can be useful to detect steroid synthesizing rare cell types in a specific immunological microenvironment. In many experiments, it has been shown that steroids may modulate the functions of immune cells by epigenetics changes. Chromosome conformation capture (3C) assay, circular chromosome conformation capture (4C), chromosome conformation capture carbon copy (5C) will be helpful to analyze the changes in genomic interactions [134–136]. Steroid hormone receptors (nuclear receptors) mainly exert their effect by binding chromatin and regulating gene expression. A recently developed method combining ChIP with selective isolation of chromatin-associated proteins followed by mass spectrometry to identify chromatin-bound partners of a protein of interest [137] is anticipated to shed light in this area.

To date, there is very little information on relationship between noncoding RNA and steroid metabolites. It would be interesting to study the correlation of noncoding RNA and steroid metabolites in immune cells in different microenvironment and disease condition by quantifying microRNA using miREIA and SplintR-qPCR technologies [138]. Localization of liganded steroid receptors in immune cells can be detected using fluorescent labeling (e.g., GFP tag) or by small nonfluorescent approaches (e.g., FlAsH-based method). To understand the molecular interactions in a cell population, NicheNet analysis from single-cell RNA seq of a cell population can be used. It will help predict cellular interactions in a cell population by linking ligand and target gene expression in the cells of that microenvironment. Immune reprogramming in a microenvironment and the influence of a particular steroid molecule can be detected by this approach [139]. RNA velocity analysis in single cells will greatly assist us in studying lineage trajectories, gene regulation, and to identify pathway activity [140].

**Fig. 6:**
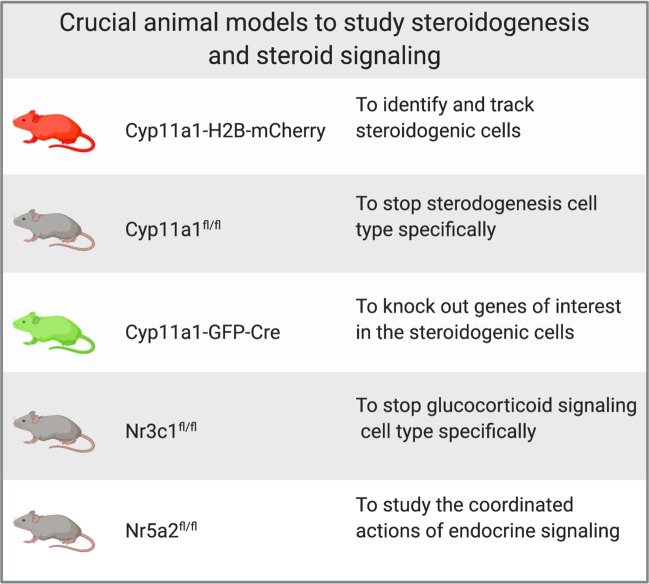
Important genetically modified mouse models that are not fully exploited to study immune-cell-mediated steroidogenesis and steroid signaling. These mice models can be instrumental to discover several aspects of endogenous steroid regulation of immune-cell function. (Figure created with BioRender.com).

There are newly developed transgenic mice that hold the potential of groundbreaking changes in the field (Fig. 6). To track steroidogenic cells in vivo, steroidogenesis reporter mice, *Cyp11a1*-H2B-mCherry reporter [12], mice can be useful. *Cyp11a1*^fl/fl^ mice can be used to delete the Cyp11a1 cell-type-specific and stop steroid biosynthesis in the cell type of interest [12]. To delete any gene of interest in steroidogenic cells *Cyp11a1*-GFP-Cre line [141] can be instrumental. To study the effect of steroids *Nr3c1*^fl/fl^ [81, 142], *Nr5a2* ^fl/fl^ [143] mice are developed, but their full potential has not been exploited.

**Fig. 7:**
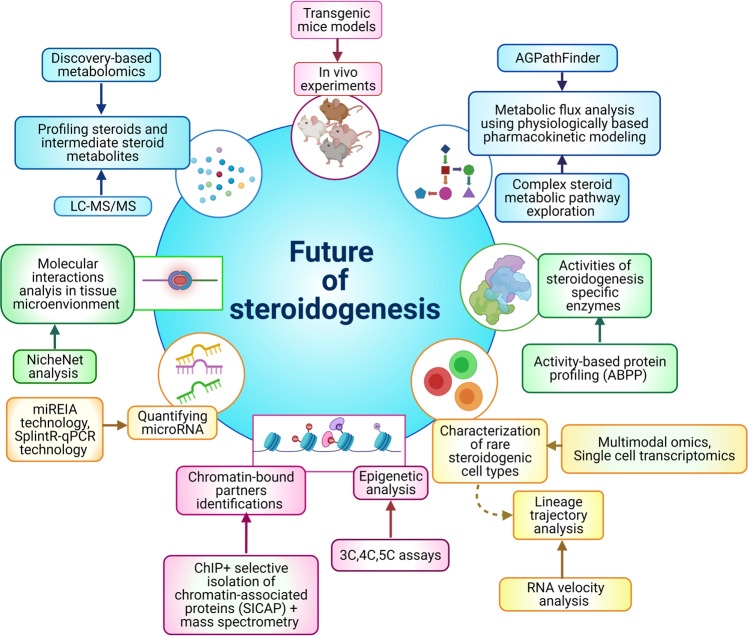
Diagrammatic summary of how technical and methodological advancement can shape the future of steroidogenesis research. This imagination reflects only authors’ viewpoint and it is likely that many other important possibilities are missed. (Figure created with BioRender.com).

Altogether, we are entering into an era full of excitement of new discoveries and innovations in the area of steroidogenesis and steroid signaling. We tried to visualize our imagination how technological and methodological advancement is anticipated to shape the future of steroidogenesis research (Fig. 7). This imagination reflects only authors’ viewpoint and it is likely that we are missing many important aspects that may bring dramatic impact on steroidogenesis research.

## Conclusion and perspectives

Steroids are indispensable biomolecules of our body. They regulate several physiological processes. Despite the myriad studies, steroid regulation of immune system is still mysterious. Steroids influence the immune-cell function based on the microenvironment, steroid type, concentration, time of exposure, and maturation stage of immune cells. Steroids have a direct or indirect influence on almost every type of immune cells, the underlying molecular mechanism of some have been explored, but mostly remain in mystery. The influence of local steroids may play a major regulatory role in these processes. Dysregulation of immune system and immune reprogramming play significant roles in the onset or progression of the disease. Steroid-producing immune cells and local steroidogenesis may play an important role in this immune regulation. Thus, exploring the influence of steroids on immune system will enrich basic science.

Synthetic steroids are used as anti-inflammatory and immunosuppressant drugs. Frequently prescribed in asthma, chronic obstructive pulmonary disease, hay fever, hive, eczema, arthritis, inflammatory bowel disease, lupus, Crohn’s disease, multiple sclerosis, organ transplantation and also in clinical oncology. Unfortunately, long-term use causes deleterious side effects and eventually, drug resistance developed. The discovery of underlying fundamental principles of immune-cell-mediated steroidogenesis and endogenous steroid-regulation of immune cells is expected to innovate novel therapeutic strategies to bypass undesirable side effects of synthetic steroids, ensuring more physiological resolution of inflammation and immunity.
